# Association of Anxiety With Pain and Disability but Not With Increased Measures of Inflammation in Adolescent Patients With Juvenile Idiopathic Arthritis

**DOI:** 10.1002/acr.24006

**Published:** 2020-07-23

**Authors:** Laura Hanns, Anna Radziszewska, Linda Suffield, Francesca Josephs, Hema Chaplin, Hannah Peckham, Debajit Sen, Deborah Christie, Livia A. Carvalho, Yiannis Ioannou

**Affiliations:** ^1^ University College London London UK; ^2^ Queen Mary University of London London UK

## Abstract

**Objective:**

To explore whether anxiety and depression are associated with clinical measures of disease for adolescent patients with juvenile idiopathic arthritis (JIA) and whether anxiety and depression are associated with increased peripheral proinflammatory cytokine levels in adolescent patients with JIA and in healthy adolescent controls.

**Methods:**

A total of 136 patients with JIA and 88 healthy controls ages 13–18 years completed questionnaires on anxiety and depressive symptoms. For patients with JIA, pain, disability, physician global assessment (using a visual analog scale [VAS]), and number of joints with active inflammation (active joint count) were recorded. In a subsample, we assessed lipopolysaccharide‐stimulated interleukin 6 (IL‐6) production from peripheral blood mononuclear cells, serum IL‐6, cortisol, and C‐reactive protein levels. Data were analyzed by linear regression analysis.

**Results:**

Levels of anxiety and depressive symptoms in patients with JIA were not significantly different than those in healthy controls. For patients with JIA, anxiety was significantly associated with disability (β = 0.009, *P* = 0.002), pain (β = 0.029, *P* = 0.011), and physician global assessment VAS (β = 0.019, *P* = 0.012), but not with active joint count (β = 0.014, *P* = 0.120). Anxiety was not associated with any laboratory measures of inflammation for JIA patients. These relationships were also true for depressive symptoms. For healthy controls, there was a trend toward an association of anxiety (but not depressive symptoms) with stimulated IL‐6 (β = 0.004, *P* = 0.052).

**Conclusion:**

Adolescent patients with JIA experience equivalent levels of anxiety and depressive symptoms as healthy adolescents. For adolescent patients with JIA, anxiety and depressive symptoms are associated with pain, disability, and physician global assessment VAS, but not with inflammation.

## INTRODUCTION

Adolescence is a time of significant physical, behavioral, and psychological change 1. Approximately one‐half of all psychiatric disorders start between late adolescence and early adulthood, and this can predict future psychopathology in adulthood 2, 3. For adolescents with a chronic disease, the changes that are normally experienced during adolescence fall alongside discovery of how to manage a chronic disease independently and making the transition from pediatric to adult care 4. With these added stressors, it is not surprising that 1 in 3 adolescents with a chronic disease are likely to experience mental health problems 5. It is therefore important to understand the psychological health of patients with a chronic disease, particularly during adolescence, with an aim of preventing current and long‐term psychiatric problems.

**Significance & Innovations**

- Adolescent patients with juvenile idiopathic arthritis (JIA) experience equivalent levels of anxiety and depressive symptoms as healthy adolescents.
- For adolescent patients with JIA, anxiety and depression are associated with pain, disability, and physician global assessment using a visual analog scale, but not with inflammation.
- Since the results of the present study show that anxiety and depression are associated with pain and disability, access to a dedicated pediatric rheumatology psychologist may benefit patients. A pediatric rheumatology psychologist could work with physical therapists and occupational health services to address pain, disability, and mental well‐being in an integrated care package for adolescents with JIA.

Juvenile idiopathic arthritis (JIA) is defined as arthritis of unknown etiology that begins before the age of 16 years and persists for a minimum of 6 weeks 6. JIA is comprised of 7 distinct categories of heterogeneous conditions as defined by the International League of Associations for Rheumatology (ILAR) 6. For adolescents newly diagnosed as having JIA, 14.7% experience high levels of depressive symptoms 7; however, the prevalence of anxiety specifically in adolescent patients with JIA is unknown.

Psychological health has been shown to be associated with disease activity in adolescent patients with JIA. We showed that for adolescents recently diagnosed with JIA, depressive symptoms were associated with disease activity at baseline and could predict future pain and disability for up to 4 years after baseline 7. The relationship between anxiety and disease activity in adolescent patients with JIA remains unclear, and existing studies in combined child and adolescent populations have demonstrated mixed results 8, 9, 10. Furthermore, none of these studies have explored potential mechanisms or pathways that might be driving an association between anxiety and disease activity.

One possible explanation for an association between anxiety and disease activity is that anxiety may be associated with an increase in peripheral inflammatory cytokines. This relationship has been shown in adults 11, 12, 13, but adolescent populations are considerably less well‐studied. Pallavi et al compared the association between anxiety and inflammation in a group of healthy adolescents and a group of adolescents with major depressive disorder (MDD) 14. In the adolescents with MDD, both state and trait anxiety were associated with lower levels of serum interleukin 17 (IL‐17) and transforming growth factor β1 (TGFβ1) and increased levels of IL‐10 and IL‐1β in females 14. However, Copeland et al 15 found no associations between anxiety and C‐reactive protein (CRP) level in a large cohort of individuals ages 9–19 years from the general population. Further studies are needed to fully establish whether there is an association between anxiety and inflammation. If anxiety is associated with increased inflammation for adolescents, then understanding this association better may be important in defining predisposition to flares of disease and formulating holistic management strategies during such flares.

This study had 3 primary aims, including comparing the levels of anxiety experienced by adolescent JIA patients and healthy adolescent controls, exploring whether anxiety is associated with clinical measures of disease for adolescent patients with JIA, and determining whether anxiety is associated with increased peripheral proinflammatory cytokine levels in both adolescent JIA patients and healthy adolescent controls.

## PATIENTS AND METHODS

#### Patient recruitment

Patients attending the University College London Hospital adolescent rheumatology outpatient clinic in London, UK were recruited if they were ages 13–18 years, had not recently been treated with steroids, and if they had 1 of the following diagnoses of JIA according to ILAR criteria: systemic onset JIA, oligoarticular JIA, extended oligoarticular JIA, polyarthritis (rheumatoid factor positive or negative), psoriatic arthritis, or enthesitis‐related arthritis. Healthy controls were recruited from local high schools if they were ages 13–18 years with no autoimmune or inflammatory disease or recent infection. Ethical approval to undertake the study was given by Harrow National Research Ethics Committee (REC 11/LO/0330). Written informed consent was obtained from parents/guardians for patients younger than 16 years of age, and the patient/healthy control provided informed assent. For patients ages 16 years or older, written informed consent from the patient/healthy control was obtained. Consent was obtained according to the Declaration of Helsinki.

#### Psychological questionnaires

Depressive symptoms were assessed at baseline using the Mood and Feelings Questionnaire (child self‐report) 16. Symptoms of overall anxiety levels, state anxiety, and trait anxiety were assessed using the State‐Trait Anxiety Inventory 17.

#### Clinical measures of disease

For patients, measures of disease were recorded from clinical notes, including the number of joints with active inflammation as assessed by the rheumatologist (active joint count), physician global assessment (on a 0–10‐cm visual analog scale [VAS]), disability (Child Health Assessment Questionnaire, completed by the patient), pain (0–10‐cm VAS, completed by the patient), medication, erythrocyte sedimentation rate (ESR), and CRP level. The Juvenile Arthritis Disease Activity Score of 3 was used as a composite measure of disease activity 18. When there was no physician global assessment VAS recorded in the patient's letter or notes, a retrospective physician global assessment VAS was generated. Two rheumatologists (including YI) who treat adolescent JIA patients were asked to independently assess the clinic visit letter and estimate a physician global assessment VAS. The average score was then used.

#### Laboratory measures of inflammation

Peripheral blood mononuclear cells (PBMCs) were separated from whole blood using density gradient separation and were cryopreserved. Serum was separated from whole blood and stored at –80°C. PBMCs were later thawed in RPMI medium plus 10% fetal bovine serum and plated at 150,000 cells per well in a flat‐bottomed 96‐well microplate. Cells were stimulated with 20 ng/ml lipopolysaccharide (Salmonella Enterica; Sigma) and incubated at 37°C and 5% CO_2_ for 24 hours. Cell culture plates were then centrifuged at 1,800 revolutions per minute for 10 minutes with brake, and then supernatants were separated and stored at –80°C. The IL‐6 levels in the culture supernatant were measured using an IL‐6 Luminex kit (Bio‐Plex Pro Human Cytokine Group I 1‐plex assay; Bio‐Rad).

Serum IL‐6 was measured using the Quantikine high sensitivity IL‐6 enzyme‐linked immunosorbent assay (ELISA) kit (R&D Systems). Serum cortisol was measured using the Cortisol ELISA kit (IBL International). For JIA patients, CRP levels were reported in their clinical notes. For healthy controls, serum CRP level was measured using CRP DuoSet ELISA kit (R&D Systems, catalog no. DY1707). Sera from JIA patients were tested to validate this ELISA kit.

#### Data analysis

Data were tested for normal distribution by Kolmogorov‐Smirnov test. Associations between demographic, psychological, clinical, and laboratory data were first analyzed using Spearman's correlation, Mann‐Whitney test, Fisher's exact test, and chi‐square test with a Z score post hoc test. Bonferroni correction was calculated as 0.05/number of tests.

Regression models were generated to explore the associations between anxiety (independent variable) and clinical measures of disease (dependent variable), as well as the associations between depressive symptoms (independent variable) and clinical measures of disease (dependent variable). The clinical measures of disease that were explored in regression models included active joint count, disability, pain, and physician global assessment VAS. A separate regression model was carried out for each clinical measure of disease against each psychological variable (anxiety or depression). Next, associations between anxiety and laboratory measures of inflammation were explored as well as associations between depressive symptoms and laboratory measures of inflammation. A separate regression model was carried out for each laboratory parameter against each psychological variable (anxiety or depression). Age and sex were controlled for in all models. For models exploring laboratory measures of inflammation, the time of day of the blood sample collection (e.g., morning, afternoon) was also controlled for. These covariates were included in the analyses regardless of significance in the Spearman's correlation and Mann‐Whitney tests. Separate models were generated for patients with JIA and healthy controls. Clinical and psychological questionnaire data were not log transformed, in order to aid direct interpretation of the clinical relevance of these common clinical measures for psychologists and rheumatologists. Laboratory measures of inflammation were log transformed to ln(measure + 1), because direct interpretation of laboratory measures in pg/ml units is not very useful for assessing the clinical relevance of these data. Data were analyzed using GraphPad Prism (version 5) and SPSS (version 21).

## RESULTS

A total of 144 patients were initially recruited to this study. Eight patients were excluded because they were being treated with oral prednisolone or had recently received intraarticular steroids, leaving a total of 136 patients in the study. A total of 88 heathy controls were recruited to this study. The demographic and psychological questionnaire data are shown in Table 1, along with the clinical measures of disease for JIA patients. Data were not normally distributed and so nonparametric tests were used. None of the demographic or psychological questionnaire scores were significantly different between patients and healthy controls.

**Table 1:** Demographic data, psychological questionnaire scores, and disease activity scores for adolescent healthy controls (n = 88) and adolescent JIA patients (n = 136)^a^

Characteristics	Adolescent healthy controls		Adolescent JIA patients	
	Median (IQR)	No. (%)	Median (IQR)	No. (%)
Age, years	16 (16–17)	88 (100)	17 (16–18)	136 (100)
Sex				
Male	–	41 (46.6)	–	65 (47.8)
Female	–	47 (53.4)	–	71 (52.2)
STAI				
Total anxiety	77 (64–88)	88 (100)	70 (55–91)	134 (98.5)
State anxiety	35.5 (28–41)	88 (100)	33 (26–43)	134 (98.5)
Trait anxiety	41 (33–49.5)	88 (100)	37.9 (28–48)	134 (98.5)
Depressive symptoms (MFQ‐C)	4 (2–7)	88 (100)	3 (1–7)	135
Subtypes				
Polyarthritis	–	–	–	45 (33.1)
Persistent oligoarthritis	–	–	–	15 (11)
Extended oligoarthritis	–	–	–	28 (20.6)
Enthesitis‐related arthritis	–	–	–	40 (29.4)
Psoriatic arthritis	–	–	–	3 (2.2)
Systemic onset JIA	–	–	–	5 (3.7)
Disease duration, years	–	–	7 (3–12)	136
Use of DMARDs				
Yes	–	–	–	77 (56.6)
No	–	–	–	59 (43.4)
Use of biologic drugs				
Yes	–	–	–	47 (34.6)
No	–	–	–	89 (65.4)
Use of NSAIDs				
Yes	–	–	–	102 (75.0)
No	–	–	–	34 (25.0)
Active joint count	–	–	0 (0–2)	128 (94.1)
Limited joint count	–	–	0 (0–2)	124 (91.1)
Physician global assessment VAS (0–10 cm)	–	–	0.5 (0–3)	121 (88.9)
Patient general VAS (0–10 cm)	–	–	1 (0–4)	114 (83.8)
Disability (C‐HAQ [0–3])	–	–	0 (0–0.5)	102 (75.0)
Pain VAS (0–10 cm)	–	–	1 (0–4)	110 (80.8)
JADAS3	–	–	1.8 (0–8.5)	110 (80.8)

a Data were compared by Mann‐Whitney, chi‐square, and Fisher's exact tests. JIA = juvenile idiopathic arthritis; IQR = interquartile range; STAI = State Trait Anxiety Inventory; MFQ‐C = Short Mood and Feelings Questionnaire, child version; DMARD = disease‐modifying antirheumatic drug; NSAID = nonsteroidal antiinflammatory drug; VAS = visual analog scale; C‐HAQ = Childhood Health Assessment Questionnaire; JADAS3 = Juvenile Arthritis Disease Activity Score 3 (active joint count plus physician global assessment VAS plus patient general VAS).

#### Associations between anxiety and clinical measures of disease for adolescent JIA patients

The associations between anxiety and clinical measures of disease were explored for adolescent JIA patients. Anxiety correlated with disability (r = 0.333, *P* < 0.001), pain (r = 0.273, *P* = 0.004), and physician global assessment VAS (r = 0.291, *P* = 0.001) but not active joint count. The regression analysis also showed anxiety to be associated with disability, pain, and physician global assessment VAS after accounting for possible confounding variables (Table 2). Anxiety was not associated with active joint count in the regression analysis. Regression analysis also showed depressive symptoms to be associated with disability, pain, and a trend toward physician global assessment VAS (but not active joint count) after accounting for possible confounding variables (see Supplementary Table 1, available on the *Arthritis Care & Research* website at http://onlin​elibr​ary.wiley.com/doi/10.1002/acr.24006/​abstract).

**Table 2:** Associations between anxiety and disease activity for adolescent JIA patients (n = 136)^a^

Dependent variable	β, unstandardized coefficient	*P,* dependent variable significance	95% CI for β, unstandardized coefficient
Active joint count (n = 126)	0.014	0.120	–0.004, 0.032
Disability (C‐HAQ) (n = 100)	0.009	0.002	0.004, 0.015
Pain (n = 108)	0.029	0.011	0.007, 0.052
Physician global assessment VAS (n = 119)	0.019	0.012	0.004, 0.034

a Data were analyzed using multiple linear regression models. Independent variable was anxiety. Age and sex were controlled for in all models. JIA = juvenile idiopathic arthritis; 95% CI = 95% confidence interval; C‐HAQ = Childhood Health Assessment Questionnaire; VAS = visual analog scale.

#### Laboratory measures of inflammation

Of the 136 patients who completed the psychological questionnaire, 100 also provided a blood sample. As not all patients donated a matching blood sample, the demographic, clinical, and psychological data of those with and without a blood sample were compared. Patients who donated a blood sample had significantly higher trait anxiety scores (median 39 [interquartile range (IQR) 29–49]) than those who did not donate a blood sample (median 32 [IQR 25–39]) (*P* = 0.008). There was also a higher proportion of patients being treated with biologics for those who provided a blood sample (40.4%) when compared to those who did not provide a blood sample (18.9%) (*P* = 0.014). Of the 88 healthy controls who completed the psychological questionnaire, 55 also provided a blood sample. There were no differences in the demographic or psychological data between healthy controls who did and did not donate a blood sample. Serum IL‐6, serum CRP level, ESR (patients only), serum cortisol, and the stimulated IL‐6 response of PBMCs were all measured (Table 3). Serum CRP levels were significantly higher for JIA patients than for healthy controls (*P* = 0.002). There were no differences in anxiety scores between healthy controls who did and did not provide a blood sample.

**Table 3:** Laboratory measures of inflammation for adolescent healthy controls (n = 88) and adolescent JIA patients (n = 100)^a^

Inflammatory measures	Adolescent healthy controls	No.	Adolescent JIA patients	No.	*P*
IL‐6 (pg/ml)	0.82 (0.43–1.27)	53	0.85 (0.52–1.62)	92	0.197
CRP (mg/liter)	0.6 (0.6–0.8)	53	0.8 (0.6–2.0)	89	0.002
ESR (mm/hour)	–	–	2 (2–7)	91	–
Cortisol (mg/ml)	68.42 (46.12–100.48)	48	59.77 (44.23–84)	87	0.338
Stimulated IL‐6 (pg/ml)	7,227.73 (3,990.78–9,605.11)	49	7,560.84 (4,528.81–11,648.37)	88	0.389

a Values are the median (interquartile range) unless indicated otherwise. C‐reactive protein (CRP) and erythrocyte sedimentation rate (ESR) data for patients with juvenile idiopathic arthritis (JIA) were collected from clinical records. IL‐6 = interleukin 6.

#### Associations between anxiety and measures of inflammation for adolescent JIA patients and healthy controls

For JIA patients, anxiety did not correlate with any laboratory measures of inflammation. Regression analysis also showed no associations between anxiety and laboratory measures of inflammation after accounting for possible confounding variables (Table 4). Similarly, there were no associations between depressive symptoms and laboratory measures of inflammation (see Supplementary Table 2, available on the *Arthritis Care & Research* website at http://onlin​elibr​ary.wiley.com/doi/10.1002/acr.24006/​abstract). For healthy controls, anxiety was correlated with stimulated IL‐6 (r = 0.295, *P* = 0.040) but not with any other laboratory measures of inflammation. The regression analysis showed that the relationship between anxiety and stimulated IL‐6 for healthy controls approached significance after accounting for possible confounding variables (Table 5). No other associations between anxiety and laboratory measures of inflammation were found in the regression analyses. No associations between depressive symptoms and laboratory measures of inflammation were found in the regression analyses for healthy controls (see Supplementary Table 3, available at http://onlin​elibr​ary.wiley.com/doi/10.1002/acr.24006/​abstract).

**Table 4:** Associations between anxiety and laboratory measures of inflammation for adolescent JIA patients (n = 136)^a^

Dependent variable	β, unstandardized coefficient	*P,* dependent variable significance	95% CI for β, unstandardized coefficient
Log serum IL‐6, pg/ml (n = 90)	0.000	0.863	–1.505, 0.637
Log serum CRP, mg/liter (n = 87)	0.001	0.702	–0.384, 0.702
Log serum cortisol, mg/ml (n = 85)	–0.001	0.363	–0.004, 0.001
Log stimulated IL‐6, pg/ml (n = 86)	0.001	0.447	–0.002, 0.004

a Data were analyzed using multiple linear regression models. Independent variable was anxiety. Age, sex, and time of day of blood sample collection (e.g., morning, afternoon) were controlled for in all models. Serum interleukin 6 (IL‐6), serum C‐reactive protein (CRP), serum cortisol, and lipopolysaccharide‐stimulated IL‐6 were log transformed; anxiety was not log transformed. JIA = juvenile idiopathic arthritis; 95% CI = 95% confidence interval.

**Table 5:** Associations between anxiety and laboratory measures of inflammation for adolescent healthy controls (n = 88)^a^

Dependent variable	β, unstandardized coefficient	*P,* dependent variable significance	95% CI for β, unstandardized coefficient
Log serum IL‐6, pg/ml (n = 53)	0.000	0.961	–0.005, 0.005
Log serum CRP, mg/liter (n = 52)	0.074	0.636	–0.003, 0.005
Log serum cortisol, mg/ml (n = 48)	0.000	0.921	–0.003, 0.003
Log stimulated IL‐6, pg/ml (n = 49)	0.004	0.052	0.000, 0.008

a Data were analyzed using multiple linear regression models. Independent variable was anxiety. Age, sex, and time of day of blood sample collection (e.g., morning, afternoon) were controlled for in all models. Serum interleukin 6 (IL‐6), serum C‐reactive protein (CRP), serum cortisol, and lipopolysaccharide‐stimulated IL‐6 were log transformed; anxiety was not log transformed. 95% CI = 95% confidence interval.

## DISCUSSION

Our study showed that adolescent patients with JIA experience the same levels of state anxiety, trait anxiety, and depressive symptoms as adolescent healthy controls. For adolescent JIA patients, both anxiety and depression were associated with disability, pain, and physician global assessment VAS but not active joint count. When laboratory measures of inflammation were explored, there was a trend toward anxiety (but not depression) being associated with increased stimulated IL‐6 levels for healthy adolescent controls. However, for adolescent JIA patients we found no associations between anxiety, depression, and laboratory measures of inflammation.

To the best of our knowledge, this is the first study to profile levels of state and trait anxiety in an exclusively adolescent population of JIA patients. We found that adolescents with JIA experience the same level of state and trait anxiety as adolescent healthy controls. These results are supported by 3 previous studies in JIA patients 8, 9, 19. However, these studies were in mixed child and adolescent populations of JIA patients and had small patient numbers. Other studies have found JIA patients to have a greater prevalence of anxiety 20, 21. We found that adolescents with JIA experience the same level of depressive symptoms as adolescent healthy controls. Previous studies have demonstrated mixed results as to whether JIA patients experience differing levels of depressive symptoms compared to healthy children 8, 9. Considering that we found an association between disability, pain, physician global assessment VAS and both anxiety and depression (discussed below), the fact that adolescent JIA patients in this study experienced equivalent levels of anxiety and depressive symptoms as healthy controls may be a reflection of the generally good control of disease activity in this population. If we had recruited only those patients experiencing a flare of disease, then we may have seen differences in the anxiety scores and depressive symptoms of adolescent JIA patients compared to healthy controls.

The association between anxiety and disease activity for adolescent JIA patients was explored next. Anxiety was not associated with active joint count, which is consistent with results of previous studies 8, 9. Anxiety was found to be associated with physician global assessment VAS, disability, and pain. Previous studies have not found an association between anxiety, physician global assessment VAS, and pain 8, 9, 10, and there have been mixed results regarding the association between anxiety and disability for patients with JIA 8, 9. All of these previous studies have used different psychological questionnaires and are from mixed populations of children and adolescents, both of which may be factors contributing to differences in findings. We found depression to be associated with disability, pain, and physician global assessment VAS (see Supplementary Table 1, available on the *Arthritis Care & Research* website at http://onlinelibrary.wiley.com/doi/10.1002/acr.24006/abstract), which is in agreement with several previous studies in pediatric JIA patients 7, 8, 9, 10, 22.

There are several considerations to make when assessing the relevance of the associations found between anxiety, low mood, physician global assessment VAS, disability, and pain. First, physician global assessment VAS, disability, and pain were all measured by questionnaires and were thus more open to bias and individual interpretation than objective measures of disease activity such as active joint count. Second, although the associations between anxiety, depression, pain, disability, and physician global assessment VAS reached statistical significance, this does not always translate into clinical significance. Very few patients in this cohort had high disease activity scores, and this cohort had well‐controlled disease activity in general. Therefore, the clinical relevance of these associations is uncertain. Another important consideration is that because of the cross‐sectional nature of this study, the directionality behind associations found remains unknown. If worsened pain and disability drives anxiety and low mood, then in this cohort with generally low disease activity levels one may not expect to see clinically relevant levels of change in psychological health. Conversely, if anxiety and low mood drive pain and disability, then the generally low disease activity levels in this cohort may preclude any clinically significant associations being found.

Associations between anxiety and inflammation were subsequently explored. No associations between anxiety and serum IL‐6 and CRP levels for either JIA patients or healthy controls were found. Similarly, no associations were found between depression and serum IL‐6 and CRP levels for either JIA patients or healthy controls. The association between depression and increased circulating proinflammatory cytokines has been well characterized in adults 23. There have been mixed results in studies of adolescents, although negative results were possibly due to the studies being underpowered 24, 25, 26, 27, 28. In comparison to the number of studies published exploring the association between depression and inflammation, very few studies have been published showing associations between anxiety and inflammation, particularly in adolescent populations 14, 15.

As expected, the results of our study showed a trend toward anxiety being associated with increased stimulated IL‐6 levels for healthy controls. It has been shown that patients with depression and anxiety have higher stimulated proinflammatory cytokine responses and that increased symptoms of anxiety and depression are associated with increased proinflammatory cytokine release 11, 29, 30. Interestingly, this association was not seen for JIA patients. The population of JIA patients recruited to this study had, on average, low disease activity scores. Adolescent JIA patients had significantly higher CRP levels than adolescent healthy controls. However, there were no other differences in the inflammatory profile of JIA patients and healthy controls. Although patients who had recently taken oral or intraarticular steroids were excluded from this study, it is possible that other antiinflammatory medications taken by patients contributed toward the lack of association between anxiety and increased laboratory measures of inflammation for JIA patients. If a population of treatment‐naive patients were studied, it is possible that an association between anxiety, low mood, and inflammation may have been found. As it stands, the present study recruited patients regardless of disease activity, medication use (other than steroids), or disease duration, and thus our findings are representative of the general adolescent JIA population.

Persistently active JIA is often associated with significant short‐term and long‐term disability. Persistent or re‐occurring synovial inflammation can lead to permanent damage of the cartilage and bone if not treated quickly and effectively 31. Pain experienced by JIA patients can also be caused by synovitis as well as joint damage, which is a common cause of noninflammatory pain 32. Noninflammatory pain, which is defined as pain that cannot be explained by active disease or inflammation, is very common in JIA patients. In a large cross‐sectional analysis of 388 JIA patients (ages 9–16 years), only 19.6% of variation in pain VAS scores could be explained by measures relating to disease activity, meaning that over 80% of pain was related to factors other than active disease 33. In addition to joint damage pain sensitization, pain‐specific beliefs and coping strategies used in response to pain all also contribute toward the pain experienced by JIA patients 34, 35. This discrepancy between active inflammation and pain for JIA patients highlights the multifaceted nature of pain.

There are many common and interconnected factors that contribute to both the pain and disability experienced by JIA patients. In the simplest sense, experiencing pain and disability can predispose patients to low mood and anxiety, and those with anxiety and low mood may be more likely to overreport symptoms of pain and disability. Furthermore, patients with anxiety and low mood may be less likely to comply with self‐management behaviors such as adherence to medication, physical therapy exercises, and attending appointments, as well as possibly being less likely to seek out practical and social support 36, 37, 38. Reduced adherence to self‐management behaviors may then result in worsened disease activity and disability. Pain and depression also share common biological processes. Noradrenaline and serotonin inhibit pain pathway signaling. Therefore, the lowered serotonin and noradrenaline levels that are associated with depression can result in increased pain for some patients 39. The true prevalence of comorbid pain and depression is unclear, and rates reported in the literature vary from 15% to 100% depending on the measures of pain and depression used, thresholds of scoring used, and patient populations 37. Thinking behaviors, such as catastrophizing, may also mediate the relationship between pain, anxiety, and depression. Adolescents who use catastrophizing as a coping strategy for pain report more depressive symptoms, which may be due to the rumination type behaviors that are common to both 40. These alterations in emotional regulation are particularly relevant for adolescents. The prefrontal cortex (responsible for self‐control) does not fully mature until adulthood, whereas the limbic system (responsible for emotional reactivity) is already fully developed 1. This results in a bias toward poor cognitive emotion regulation, which in turn is associated with symptoms of depression 1, 41. The pain experienced by JIA patients can also lead to reduced physical activity 42. This decreased activity may lead to a cycle of physical deconditioning, worsened disability, and worsened pain, particularly for patients who believe that exercising will cause further joint damage, disability, and pain 42, 43. The social withdrawal associated with anxiety and depression is also likely to contribute to reduced physical activity and exacerbate this cycle 42, 44.

In the present study, the fact that pain and disability were associated with anxiety and depression but not active joint count or laboratory measures of inflammation suggests that for adolescent JIA patients, anxiety and depression may be more strongly associated with noninflammatory pain and disability caused by factors other than current synovitis. However, patients in this study generally had well‐controlled disease activity, with only a limited number of patients experiencing clinically significant levels of inflammation. This may have precluded any associations being found between inflammation, pain, and disability in this cohort.

The present study has found cross‐sectional associations between anxiety, depression, pain, and disability for adolescent JIA patients. However, the underlying directionality remains unknown. Increased psychological support at diagnosis may lead to improvements in pain and disability. It is also equally possible that treating pain and disability may reduce anxiety and depressive symptoms, negating the need for extra psychological support. Moreover, bidirectional and interconnecting factors may mean that both of these statements are true. A prospective longitudinal study to assess the impact of increased psychological support on pain, disability, and mental well‐being in recently diagnosed adolescent JIA patients is warranted. A dedicated pediatric rheumatology psychologist could focus on treating the known psychological components of pain and disability as well as mental well‐being. This increased psychological support should be fully integrated into the existing multidisciplinary clinical care package that patients currently receive. A psychological intervention study would also help to determine the directionality of the associations between pain, disability, and mental well‐being.

There were also potential limitations to the present study. Age and sex were included in our regression analyses as covariates. If a much larger population of adolescent JIA patients had been recruited, other variables known to be associated with disease activity could have been accounted for, such as medication use, JIA subtype, socioeconomic status, and disease duration 6, 45, 46. The present study used the pain VAS as a measure of pain, as it is widely used both in clinical practice and in academic studies. However, the 0–10‐cm scale of the pain VAS cannot provide information about arthritis‐specific pain, pain intensity, frequency, pain sensation, or the location of the pain. Therefore, for future studies, a multidimensional pain tool such as E‐Ouch, an electronic pain diary developed specifically for adolescent JIA patients, should be used instead of the pain VAS 47.

In conclusion, this detailed analysis across a broad population of adolescents with JIA treated in the postbiologic era has shown that this group of patients experiences equivalent levels of state anxiety, trait anxiety, and depressive symptoms as healthy adolescents. However, pain, disability, and physician global assessment VAS scores were found to be associated with anxiety and depressive symptoms. Interestingly, anxiety and depressive symptoms were not associated with inflammatory measures such as active joint count, serum IL‐6 level, and CRP level. These results suggest that in this cohort, associations between anxiety, mood, pain, and disability are less likely to be mediated by inflammation and are more likely to be associated with other cognitive and behavioral mechanisms.

## AUTHOR CONTRIBUTIONS

All authors were involved in drafting the article or revising it critically for important intellectual content, and all authors approved the final version to be submitted for publication. Dr. Ioannou had full access to all of the data in the study and takes responsibility for the integrity of the data and the accuracy of the data analysis.

### Study conception and design

Hanns, Christie, Carvalho, Ioannou.

### Acquisition of data

Hanns, Radziszewska, Suffield, Josephs, Chaplin, Peckham, Sen, Christie, Carvalho, Ioannou.

### Analysis and interpretation of data

Hanns, Sen, Christie, Carvalho, Ioannou.

## ADDITIONAL DISCLOSURES

Authors Hanns and Ioannou are currently employees of UCB Celltech but were employed by University College London during the time the study was conducted.
