# Supplementary material for: Association of Anxiety With Pain and Disability but Not With Increased Measures of Inflammation in Adolescent Patients With Juvenile Idiopathic Arthritis
Source: Arthritis Care Res (Hoboken). 2020 Jul 23;72(9):1266–74. doi: 10.1002/acr.24006 (PMC7496487; doi:10.1002/acr.24006)
Supplement: Supplementary file 1 [file ACR-72-1266-s001.docx]

**Supplementary Table 1. Associations between depressive symptoms and disease activity for adolescent JIA patients**

| Dependent variable | Unstandardised β | Dependent variable significance  (p value) | Lower 95% CI for unstandardised β | Upper 95% CI for unstandardised β |
| --- | --- | --- | --- | --- |
| Active joint count  N= 126 | 0.063 | 0.081 | 0.008 | 0.135 |
| Disability (CHAQ)  N= 100 | 0.048 | <0.001 | 0.023 | 0.073 |
| Pain  N= 108 | 0.133 | 0.004 | 0.044 | 0.221 |
| Physician VAS  N= 119 | 0.058 | 0.066 | -0.004 | 0.120 |

Data were analysed using multiple linear regression models. Independent variable was depressive symptoms. Age and gender were controlled for. N=136 adolescent JIA patients.

*Childhood Health Assessment Questionnaire (CHAQ), visual analogue scale (VAS).*
