# Supplementary material for: Association of Anxiety With Pain and Disability but Not With Increased Measures of Inflammation in Adolescent Patients With Juvenile Idiopathic Arthritis
Source: Arthritis Care Res (Hoboken). 2020 Jul 23;72(9):1266–74. doi: 10.1002/acr.24006 (PMC7496487; doi:10.1002/acr.24006)
Supplement: Supplementary file 2 [file ACR-72-1266-s002.docx]

**Supplementary Table 2. Associations between depressive symptoms and laboratory measures of inflammation for adolescent JIA patients**

| Dependent variable | Unstandardised β | Dependent variable significance  (p value) | Lower 95% CI for unstandardised β | Upper 95% CI for unstandardised β |
| --- | --- | --- | --- | --- |
| Log serum IL-6 (pg/ml)  N=90 | 0.003 | 0.674 | -0.011 | 0.017 |
| Log serum CRP (mg/L)  N= 88 | 0.009 | 0.297 | -0.008 | 0.025 |
| Log serum cortisol (mg/ml)  N= 86 | -0.003 | 0.599 | -0.013 | 0.008 |
| Log stimulated  IL-6 (pg/ml)  N= 87 | 0.005 | 0.359 | -0.006 | 0.016 |

Data were analysed using multiple linear regression models. Independent variable was depressive symptoms. Age, gender and time of blood sample were controlled for. Serum IL-6, serum CRP, serum cortisol and stimulated IL-6 were log transformed. Depressive symptoms score was not log transformed. N=136 adolescent JIA patients.

*C reactive protein (CRP), erythrocyte sedimentation rate (ESR).*
