# Supplementary material for: Association of Anxiety With Pain and Disability but Not With Increased Measures of Inflammation in Adolescent Patients With Juvenile Idiopathic Arthritis
Source: Arthritis Care Res (Hoboken). 2020 Jul 23;72(9):1266–74. doi: 10.1002/acr.24006 (PMC7496487; doi:10.1002/acr.24006)
Supplement: Supplementary file 3 [file ACR-72-1266-s003.docx]

**Supplementary Table 3.** **Associations between depressive symptoms and laboratory measures of inflammation for adolescent healthy controls**

| Dependent variable | Unstandardised β | Dependent variable significance  (p value) | Lower 95% CI for unstandardised β | Upper 95% CI for unstandardised β |
| --- | --- | --- | --- | --- |
| Log serum IL-6 (pg/ml)  N=52 | 0.008 | 0.424 | -0.012 | 0.029 |
| Log serum CRP (mg/L)  N=52 | 0.008 | 0.340 | -0.009 | 0.024 |
| Log serum cortisol (mg/ml)  N=48 | -0.005 | 0.403 | -0.017 | 0.007 |
| Log stimulated IL-6 (pg/ml)  N=49 | 0.015 | 0.082 | -0.002 | 0.031 |

Data were analysed using multiple linear regression models. Independent variable was depressive symptoms. Age, gender and time of blood sample were controlled for. Serum IL-6, serum CRP, serum cortisol and LPS-stimulated IL-6 were log transformed. Depressive symptoms score was not log transformed. N=88 adolescent healthy controls.

*C reactive protein (CRP), erythrocyte sedimentation rate (ESR), lipopolysaccharide (LPS).*
